# Cognitive impairment in Chinese traumatic brain injury patients: from challenge to future perspectives

**DOI:** 10.3389/fnins.2024.1361832

**Published:** 2024-03-11

**Authors:** Tao Liu, Shaohui Yu, Mingqi Liu, Zhihao Zhao, Jiangyuan Yuan, Zhuang Sha, Xuanhui Liu, Yu Qian, Meng Nie, Rongcai Jiang

**Affiliations:** ^1^Department of Neurosurgery, Tianjin Medical University General Hospital, Tianjin, China; ^2^Tianjin Neurological Institute, Key Laboratory of Post Neuro-Injury Neuro-Repair and Regeneration in Central Nervous System, Ministry of Education, State Key Laboratory of Experimental Hematology, Tianjin Medical University General Hospital, Tianjin, China; ^3^The George Institute for Global Health, Faculty of Medicine, University of New South Wales, Sydney, NSW, Australia

**Keywords:** traumatic brain injury, cognitive impairment, dysfunction, epidemiology, risk factors, challenges, opportunities

## Abstract

Traumatic Brain Injury (TBI) is a prevalent form of neurological damage that may induce varying degrees of cognitive dysfunction in patients, consequently impacting their quality of life and social functioning. This article provides a mini review of the epidemiology in Chinese TBI patients and etiology of cognitive impairment. It analyzes the risk factors of cognitive impairment, discusses current management strategies for cognitive dysfunction in Chinese TBI patients, and summarizes the strengths and limitations of primary testing tools for TBI-related cognitive functions. Furthermore, the article offers a prospective analysis of future challenges and opportunities. Its objective is to contribute as a reference for the prevention and management of cognitive dysfunction in Chinese TBI patients.

## 1 Introduction

Traumatic brain injury (TBI) poses a global health challenge associated with high mortality and disability rates among populations (Maas et al., 2017). As a “silent epidemic,” it is estimated that approximately 70 million people worldwide experience TBI each year, imposing significant economic burdens on both society and families (Ponsford et al., 2022). Despite being a subject of considerable attention, the magnitude of its impact is often underestimated. The patients included in hospital statistics represent only a fraction of the TBI population, and the actual incidence and burden of TBI far exceed public expectations (Jiang et al., 2019).

Mild TBI (m-TBI) is the most common type, accounting for approximately 75–90% of all TBIs. Its symptoms manifest as non-specific, including headaches, anxiety, irritability, fatigue, depression, and cognitive impairment (Fehily and Fitzgerald, 2017; Madhok et al., 2022). Despite clear evidence indicating that m-TBI can lead to long-term physiological changes, the number of individuals with prolonged m-TBI-related symptoms, especially those involving cognitive impairment, is relatively low (McInnes et al., 2017). Cognitive dysfunction in TBI patients not only adversely affects daily life, learning, and work capabilities but also increases psychological stress and a sense of social isolation, significantly reducing the overall quality of life and social functioning (Mac Donald et al., 2017; Schneider et al., 2022). Therefore, the prevention and management of cognitive dysfunction in TBI patients have become a critically important clinical and societal undertaking.

The prevention and management of cognitive dysfunction in Chinese TBI patients face numerous challenges. The primary reason is that the occurrence and recovery of cognitive impairment in TBI patients are influenced by various factors (Jiang et al., 2019; Gao et al., 2020). Evaluating the neurobehavioral sequelae of TBI patients requires a detailed understanding of medical history and a careful assessment of potential risk factors, posing a highly challenging task for frontline clinical physicians burdened with heavy workloads. Currently, our efforts still have many shortcomings, such as a lack of effective early screening and intervention measures, the absence of comprehensive personalized assessment and treatment plans, a shortage of unified epidemiological data, and the need for strengthened multidisciplinary collaboration and tracking management (Jiang et al., 2019). To improve the current situation of cognitive dysfunction in Chinese TBI patients, it is essential to systematically analyze and discuss aspects like epidemiology, etiology, risk factors, and management strategies, providing a basis and guidance for the development of scientifically sound prevention and treatment measures.

## 2 Epidemiology in Chinese TBI patients and potential etiology of cognitive impairment: is it a challenge for us?

### 2.1 Epidemiology in Chinese TBI patients

Due to differences in factors such as etiology, injury location, type, and severity of TBI, there are significant variations in the occurrence rate and baseline characteristics of cognitive dysfunction among TBI patients. Additionally, it is scientifically unsound to compare epidemiological data across different regions and populations because current studies employ diverse methods and assessment criteria. There is still a lack of standardized tools and criteria for assessing and diagnosing cognitive dysfunction in TBI patients. We are aware that TBI can result in acute neurological deficits during the initial phase and can lead to various complications in the chronic phase, with cognitive dysfunction being the most common and severe. However, the non-specific manifestations of cognitive dysfunction are diverse and challenging to diagnose. These may include declines or losses in cognitive domains such as memory, attention, executive function, language, and visual-spatial abilities, which may impact the daily life, work, study, and social interactions of patients. The relative scarcity of epidemiological studies on cognitive dysfunction in Chinese TBI patients may be attributed to the difficulty in diagnosing these diverse and non-specific manifestations. Furthermore, most studies are single-center, small-sample studies, lacking nationwide large-scale multicenter cohort studies.

To date, the largest-scale clinical characteristics and outcomes study of TBI patients in China, known as CENTER-TBI, was conducted by Gao et al. (2020). This prospective, longitudinal observational study took place across multiple centers in China and included 13,627 cases of TBI from 56 hospitals in 22 provinces. The aim was to depict the epidemiological pattern of contemporary TBI in China. The study revealed that TBI in China predominantly occurs in young and middle-aged males between the ages of 18 and 65, with the primary cause being traffic accidents. The overall mortality rate was 5%, which was lower than the expected value predicted by the CRASH prognostic model (Perel et al., 2008; Lingsma et al., 2011). Furthermore, substantial differences were identified in the treatment methods for TBI across various hospitals and regions. The study suggests that these findings provide an opportunity for comparative effectiveness research to identify optimal practices for TBI treatment and underscore the necessity of improving TBI systems in different regions. Despite the substantial TBI burden, no FDA-approved therapeutics currently exist due to an incomplete understanding of the mechanisms underlying persistent cognitive dysfunction and post-injury degeneration.

### 2.2 Potential etiology of cognitive impairment

Traumatic Brain Injury resulting from external head impact triggers a series of neuroinflammatory and pathophysiological events leading to neuropsychiatric disorders, affecting cognitive function in 15–30% of TBI patients (Jackson et al., 2004; Himanen et al., 2006; Fleminger, 2008; Till et al., 2008; Silver et al., 2009; Wang et al., 2012). Cognitive impairment in TBI patients is a complex, multifactorial process involving several potential mechanisms. Structural damage to brain tissue, especially in functional areas like the frontal lobe and hippocampus, emerges as a primary cause of cognitive dysfunction after brain trauma (Peters et al., 2009). Research showed that damage to the right hemisphere causes more severe cognitive impairment, with damage to the frontal lobe being particularly prominent (Fletcher and Henson, 2001). The neurotransmitter 5-HT, crucial for memory regulation and brain function maintenance, has shown significance in TBI research. Kline et al. discovered that selective 5-HT1A receptor agonists can reduce neuronal damage in the cerebral cortex and hippocampus, suggesting a potential avenue for therapeutic intervention in a rat TBI model (Meneses, 2003). Additionally, Sen et al. (2017) found that endoplasmic reticulum stress-activated PKR-like ER kinase (PERK) phosphorylates CREB and PSD95 proteins, reducing BDNF levels and impairing synaptic structure and function, ultimately affecting memory. This study suggests that inhibiting PERK phosphorylation may protect dendritic spines and synapses, presenting a potential therapeutic strategy. Recently, another team found that aberrant production of TDP-43 is a key factor in promoting AD neuropathology and synaptic and cognitive deterioration in mouse models of mild closed head injury (CHI). They show that excessive production of TDP-43 either resulting from a single mild CHI or from repeated mild CHI in WT mice is an important mechanism common to both AD and TBI-induced AD-like neurodegenerative disease (Gao et al., 2022). This drives our strategy to limit TDP-43 overproduction to potentially provide therapeutic approaches to prevent the development of TBI-induced AD neuropathology. Recent research also has increasingly focused on the significant role of microglia in cognitive impairment post-TBI (Willis et al., 2020; Krukowski et al., 2021; Bray et al., 2022; Packer et al., 2024). Common findings across these studies suggest that regulating microglia can reduce cognitive impairment after TBI, hinting at microglia as a potential breakthrough in future treatments for cognitive dysfunction post-TBI.

Since the first description of the meningeal lymphatic vessels (MLVs), their unique anatomical and physiological features have been gradually revealed (Louveau et al., 2015; Antila et al., 2017). Exciting recent work revealed MLVs play an important drainage pathway in the elimination of neurotoxic substances, including Aβ aggregates, extracellular tau, and alpha-synuclein aggregates in mice. Moreover, Recent studies have also revealed that MLVs play an important role in various neurological diseases, including AD, Parkinson’s disease (PD), and glioblastoma (Da Mesquita et al., 2018; Song et al., 2020; Ding et al., 2021). Great progress has also been made in the critical role of the meningeal lymphatic system in the pathogenesis of TBI (Bolte et al., 2020; Liu et al., 2023). Bolte etc. revealed even mild head trauma can lead to prominent defects in the drainage of fluorescent beads via the meningeal lymphatic system (Bolte et al., 2020). Mouse studies from our group indicate that exogenous IL-33 exert a protective effect on cognitive functions and improve the drainage of MLVs to deep cervical after TBI (Liu et al., 2023). These studies suggest that cognitive impairment after TBI may be closely related to disruption of meningeal lymphatic drainage. This provides new avenues for future intervention in cognitive impairment after brain trauma.

## 3 Risk factors of cognitive impairment in Chinese TBI patients: do we need early intervention?

The risk factors for cognitive impairment in TBI patients, that can be divided into two categories: injury-related factors and individual-related factors, are multifaceted, requiring a comprehensive consideration of injury-related and individual-related factors and their interactions (Himanen et al., 2011; Nordström et al., 2013; Mendez, 2017; Kachmar et al., 2018). Research found that early intervention in TBI can significantly improve outcomes (Robertson et al., 2015; Laing et al., 2022). Therefore, this section will introduce the risk factors for cognitive impairment in TBI patients, to timely identify and intervene, and promote patient recovery and improve prognosis.

### 3.1 Injury-related factors

It includes factors such as the type, severity, location, and frequency of the injury, determining the extent and severity of brain damage as well as secondary damages like bleeding, hypoxia, inflammatory responses, edema, and ischemia following brain injury (Mendez, 2017; Ma et al., 2019).

Types of injuries can be classified into open and closed injuries. Open TBIs are associated with a higher incidence of coagulopathies, which may exacerbate bleeding and further increase the morbidity and mortality rates of TBI (Chen et al., 2021). Compared to open TBIs, closed injuries often result in mild TBI (Nokkari et al., 2015).

The severity of the injury can be assessed based on indicators such as duration of loss of consciousness, increased intracranial pressure, and abnormalities in neuroimaging. Generally, the higher the severity of the injury, the greater the extent and range of brain damage, and the higher the risk and degree of cognitive impairment (Lennon et al., 2023). Cognitive deficits resulting from mild TBI mostly recover completely within 3–6 months (Rabinowitz and Levin, 2014), whereas up to 65% of patients with moderate to severe TBI suffer from long-term cognitive impairments (Whiteneck et al., 2004).

The location of the injury can affect different cognitive domains. Generally, the frontal and temporal lobes are critical structures for cognitive functions, and damage to these areas can lead to cognitive and social dysfunction (Cristofori and Levin, 2015). Lesions in the left temporal lobe can cause a decline in conversational speech and auditory comprehension, lesions in the left frontal lobe can result in reduced verbal fluency, and lesions in the right parietal lobe can lead to decreased auditory comprehension and reasoning abilities (Gauthier et al., 2018).

The frequency of injuries reflects the cumulative effects of brain damage, where multiple TBIs lead to more severe brain damage and cognitive impairment, with slower and more difficult recovery in patients with multiple TBIs (Lennon et al., 2023). An animal study showed that repeated mild TBIs led to cognitive dysfunction in mice during both acute and chronic phases, along with changes in the expression of genes related to inflammation and excitotoxicity. Especially in mice subjected to 15 impacts, compared to the control group, their hippocampal spatial learning and memory abilities were impaired in the acute phase, with this damage persisting into the chronic phase (Hiskens et al., 2021).

### 3.2 Individual-related factors

Individual-related factors refer to those associated with the patient themselves, such as age, gender, baseline cognitive level, genetic factors, and comorbidities. These factors affect the sensitivity and plasticity of brain injury, as well as the capacity for repair and reorganization post-injury. Different individual-related factors may impact various cognitive domains differently, and there may be interactions between these factors, making the risk and degree of cognitive impairment more difficult to predict and assess (Himanen et al., 2011; Skaansar et al., 2020; Gomez et al., 2021; Mollayeva et al., 2021; Izzy et al., 2022; Kennedy et al., 2022; Mair et al., 2022; Hume et al., 2023).

Age is a significant factor affecting cognitive impairment, generally, the older the age, the higher the risk and degree of cognitive impairment (Hukkelhoven et al., 2006). Elderly patients with TBI, despite higher initial GCS scores, still have higher overall mortality and disability rates. This adverse prognosis may not only stem from the biological aging process and pre-existing comorbidities but also be influenced by medical decision-making biases, such as more conservative or limited diagnostic and therapeutic measures for elderly patients due to anticipated poor treatment outcomes (Skaansar et al., 2020).

Gender is also a factor affecting cognitive impairment. This may be related to differences in brain structure and function between males and females, as well as the role of sex hormones (Daneshvar et al., 2011). Compared to males, female patients have poorer cognitive recovery at 6 months, and more severe symptoms of depression and anxiety (Moretti et al., 2012).

Baseline cognitive level refers to the cognitive abilities of a patient before the occurrence of TBI. Increasing or maintaining cognitive reserve may help prevent the exacerbation of cognitive decline in the elderly following TBI (Moretti et al., 2012). TBI patients without education have worse cognitive performance compared to those with education, and a higher level of education may help to delay or mitigate the decline in cognitive functions post-TBI (Sharbafshaaer, 2018).

Genetic factors refer to those associated with the genes of TBI patients, which can influence the sensitivity and plasticity of brain injury, as well as the capacity for post-injury repair and reorganization, thereby affecting the risk and severity of cognitive impairments. Studies have shown that certain genetic variations, such as APOE ε4, result in TBI patients carrying the ε4 allele being more prone to adverse outcomes (Zhou et al., 2008; McFadyen et al., 2021). Studies on other genetic variations, like BDNF, indicate that individuals under 45 years old carrying the rs6265 Val (Val66Met) homozygote and rs712444 T allele have the highest survival probabilities in the subacute phase, suggesting that these genes may reduce the risk and severity of cognitive impairments in TBI patients (Failla et al., 2015). The GRIN genotype may affect the plasticity and cognitive function recovery post-TBI, leading to cognitive impairments (Raymont et al., 2008).

Comorbidities refer to other diseases or symptoms that occur concurrently with or subsequent to TBI, such as depression, anxiety, insomnia, pain, diabetes, hypertension, etc. These comorbidities can affect the daily living activities of TBI patients, as well as their abilities in communication, perception, thinking, reasoning, and memory, thereby impacting their overall rehabilitation progress and quality of life (Mollayeva et al., 2017; Hanafy et al., 2021). Additionally, post-concussion syndrome (PCS) that develops after TBI may have lasting effects on cognitive, memory, learning, and executive functions (McInnes et al., 2017).

## 4 Management strategies for cognitive dysfunction in Chinese TBI patients

Cognitive dysfunction is a common complication in TBI patients, significantly impacting their quality of life and social functioning. Therefore, effective cognitive function assessment and rehabilitation intervention are necessary for TBI patients. This section will introduce management strategies for cognitive dysfunction in Chinese TBI patients, including assessment methods, intervention measures.

### 4.1 Assessment methods

As the foundation of cognitive dysfunction management, the purpose of cognitive function assessment is to determine the patient’s level of cognitive function, the extent of impairment, and the scope of impact, providing a basis for the formulation of intervention plans and the evaluation of outcomes (Woodford and George, 2007).

Neuropsychological assessment, traditionally used to evaluate the degree of impairment in specific skills and identify potentially affected brain regions, focuses on assessing cognition and behavior, with a crucial emphasis on evaluating the patient’s mental state (Sun et al., 2017). The currently validated cognitive function assessment tools include the Mini-Mental State Examination (MMSE), Montreal Cognitive Assessment (MoCA), Neurobehavioral Cognitive Status Examination (NCSE), Cognitive Abilities Screening Instrument (CASI), Loewenstein Occupational Therapy Cognitive Assessment (LOTCA) (Mitrushina et al., 1994; Teng et al., 1994; Nasreddine et al., 2005; Mitchell, 2009; Almomani et al., 2018). The main advantages and disadvantages are summarized in Table 1.

**TABLE 1:** Summary of the major cognitive function assessment tools in TBI.

Testing tool	Advantages	Disadvantages
MMSE	Simple operation, applicable to different cultures and education levels	Low sensitivity, easily influenced by age, education level, and sensory factors
MoCA	High specificity and sensitivity for mild cognitive impairment	Strong dependence on culture and education level, time-consuming
NCSE	Low false negative rate	Influenced by age and cultural background, time-consuming
CASI	Not restricted by cultural level	Slightly longer testing time
LOTCA	Applicable to patients of all ages and cognitive levels, assesses multiple cognitive domains	Testing may be influenced by language and cultural background

MMSE, mini-mental state examination; MoCA, Montreal cognitive assessment; NCSE, neurobehavioral cognitive status examination; CASI, cognitive abilities screening instrument; LOTCA, Loewenstein occupational therapy cognitive assessment.

Electrophysiological markers, including electroencephalogram (EEG), sensory-evoked potentials (EPs), and event-related potentials (ERPs), can be used for electrophysiological diagnosis and monitoring of brain function (Amantini et al., 2005; Carrai et al., 2010; Dockree and Robertson, 2011). Valuable indicators such as P300 and mismatch negativity (MMN) are included (Zarza-Luciáñez et al., 2007; Kodama et al., 2010).

Brain imaging methods, such as computerized tomography (CT) and magnetic resonance imaging (MRI), provide essential information, especially for moderate to severe TBI. Diffusion tensor imaging (DTI) can measure changes in brain microstructure and white matter connectivity related to TBI (Hashim et al., 2017; Sun et al., 2017; Königs et al., 2018).

Genetic polymorphisms, such as Brain-derived neurotrophic factor (BDNF), 5-HTTLPR, and the functional catechol-*O*-methyltransferase genotype (rs4680), are associated with cognitive and social function recovery after TBI (Krueger et al., 2011; Kurowski et al., 2016).

### 4.2 Intervention measures

Currently, interventions for cognitive impairment in patients after TBI mainly include the following aspects:

#### 4.2.1 Pharmacological treatment

Pharmacological treatment primarily aims to improve brain metabolism, promote the recovery of neuronal function, inhibit apoptosis, and increase neurotrophic factors to enhance cognitive function (Togher et al., 2014). Commonly used medications include acetylcholinesterase inhibitors, serotonin receptor agonists, hormone replacement therapy, but there is a lack of large-scale randomized controlled trials and long-term follow-up studies (Togher et al., 2014; Shih et al., 2019; Ponsford et al., 2022). Therefore, the effectiveness and safety of pharmacological treatment require further validation.

#### 4.2.2 Rehabilitation training

Rehabilitation training refers to stimulating and training the impaired cognitive functions of patients through cognitive tasks, aiming to improve their cognitive levels and adaptability. Currently, several studies have shown significant improvement in cognitive function in TBI patients through rehabilitation training, and the training effects have a certain degree of sustainability and transferability (Jeffay et al., 2023). However, there is no uniform standard or guideline for the optimal timing, frequency, and intensity of rehabilitation training. Therefore, personalized rehabilitation training plans need to be developed based on the specific circumstances of each patient and individual differences.

#### 4.2.3 Other intervention measures

In addition to pharmacological treatment and rehabilitation training, there are other intervention measures that may have a certain impact on the cognitive function of TBI patients, such as nutritional supplementation, psychological support, and social engagement (Tate et al., 2014; Bayley et al., 2023; Togher et al., 2023). While these interventions may not directly target cognitive function, they could potentially influence cognitive function indirectly by improving the patient’s physical, psychological, and social health (Gómez-de-Regil et al., 2019; Coxe et al., 2021). Currently, more evidence is needed to support the effectiveness and applicability of these intervention measures.

## 5 New challenges and opportunities

With the development of society and technological progress, the management of cognitive dysfunction in Chinese TBI patients faces new challenges and opportunities. For instance, factors such as population aging and an increase in incidents like traffic accidents, violent events, and sports injuries may contribute to a higher incidence of TBI, leading to a rise in the occurrence of cognitive dysfunction among TBI patients. As medical capabilities improve, the survival rate and lifespan of TBI patients may increase, resulting in a larger population of TBI patients. The advancement of cognitive neuroscience will require a more refined and precise understanding of the pathological mechanisms and classifications of cognitive dysfunction in TBI patients, demanding more sophisticated and specialized diagnostic and assessment tools and methods.

As patient needs become more diverse, the diagnosis and assessment of cognitive dysfunction in TBI patients may require more comprehensive and personalized evaluation metrics and content. With increasing patient expectations, the intervention and management of cognitive dysfunction in TBI patients may necessitate more effective and safe intervention methods and technologies. Additionally, the intervention and management of cognitive dysfunction in TBI patients may face additional challenges and difficulties related to economic, legal, ethical, cultural, and other societal pressures.

However, with the emergence of new technologies and methods, along with the development of information technology, the prevention and intervention of cognitive dysfunction in TBI patients are becoming more personalized and intelligent. The assessment and monitoring of cognitive dysfunction in TBI patients are becoming more convenient and efficient, providing additional avenues and means for the diagnosis and care of cognitive dysfunction in TBI patients.

## 6 Future perspectives

With the development of society and technological progress, the management of cognitive dysfunction in Chinese TBI patients is facing new challenges and opportunities. Particularly, recent breakthroughs in modern medicine and life sciences have extended people’s lifespan also aggravates aging, potentially increasing the incidence of TBI in the elderly and making cognitive dysfunction issues more prominent. Additionally, the rise in incidents such as traffic accidents, violence, and sports-related activities may contribute to an increased occurrence of TBI, leading to a higher prevalence of cognitive dysfunction in TBI patients. However, limited medical resources may pose challenges to early diagnosis and proactive treatment of cognitive dysfunction in TBI patients. What’s more, as societal pressures increase, interventions and management of cognitive dysfunction in TBI patients may encounter additional economic, legal, ethical, and cultural challenges and difficulties. Nevertheless, these challenges also present opportunities for the management of cognitive dysfunction in TBI. With technological advancements, the emergence of new techniques and methods, the understanding of the pathological mechanisms and classification of cognitive dysfunction in TBI patients has become more refined and precise. Prevention and intervention are becoming more personalized and intelligent.

As patient expectations rise and demands become more diverse, and with clinical physicians focusing on long-term follow-ups and outcomes related to the family and social functions of TBI patients, there is potential to drive the development of comprehensive rehabilitation models and service systems. Furthermore, the development of cognitive neuroscience holds promise in providing new insights and tools for the diagnosis, prediction, and treatment of cognitive dysfunction in TBI patients.

## 7 Conclusion

In this article, we have conducted a review and analysis of the epidemiology, underlying mechanisms, influencing factors, assessment methodologies, and management strategies pertaining to cognitive impairment in Chinese patients with TBI. This encompasses a synthesis of the current challenges and future development trends and opportunities in this field. We posit that our article offers substantive guidance for enhancing clinical research and management practices related to cognitive impairment in TBI patients within the Chinese context. Nonetheless, our review acknowledges certain limitations, most notably the absence of large-scale epidemiological surveys on the incidence of cognitive impairment in TBI. This gap is attributed to the lack of standardized research methodologies and assessment criteria, leading to significant variations in study outcomes and the difficulty in drawing uniform conclusions. Future research endeavors should focus on more systematic and standardized investigations to provide a robust foundation for the prevention and treatment of cognitive impairment in TBI patients.

## Author contributions

TL: Conceptualization, Investigation, Methodology, Supervision, Writing – original draft, Writing – review and editing. SY: Conceptualization, Investigation, Writing – original draft. ML: Conceptualization, Methodology, Writing – original draft. ZZ: Conceptualization, Investigation, Methodology, Writing – original draft. JY: Conceptualization, Investigation, Methodology, Writing – original draft. ZS: Conceptualization, Investigation, Methodology, Writing – original draft. XL: Investigation, Writing – original draft. YQ: Investigation, Supervision, Writing – original draft. MN: Conceptualization, Investigation, Supervision, Writing – original draft, Writing – review and editing. RJ: Conceptualization, Investigation, Methodology, Supervision, Writing – original draft, Writing – review and editing.
